# Studying Abnormal Chromosomal Diseases Using Patient-Derived Induced Pluripotent Stem Cells

**DOI:** 10.3389/fncel.2020.00224

**Published:** 2020-08-13

**Authors:** Yohei Hayashi, Miho Takami, Mami Matsuo-Takasaki

**Affiliations:** iPS Advanced Characterization and Development Team, RIKEN BioResource Research Center, Tsukuba, Japan

**Keywords:** abnormal chromosome, iPSCs, chromosomal deletion, trisomy, monosomy

## Abstract

Chromosomal abnormality causes congenital and acquired intractable diseases. In general, there are no fundamental treatments for these diseases. To establish platforms to develop therapeutics for these diseases, patient-derived induced pluripotent stem cells (iPSCs) are highly beneficial. To study abnormal chromosomal diseases, it is often hard to apply animal disease models because the chromosomal structures are variable among species. It is also difficult to apply simple genome editing technology in cells or individuals for abnormal chromosomes. Thus, these patient-derived iPSCs have advantages for developing disease models with multiple cell and tissue types, which are typically seen in the symptoms of abnormal chromosomal diseases. Here we review the studies of patient-derived iPSCs carrying abnormal chromosomes, focusing on pluripotent state and neural lineages. We also discuss the technological advances in chromosomal manipulations toward establishing experimental models and future therapeutics. Patient-derived iPSCs carrying chromosomal abnormality are valuable as cellular bioresources since they can indefinitely proliferate and provide various cell types. Also, these findings and technologies are important for future studies on elucidating pathogenesis, drug development, regenerative medicine, and gene therapy for abnormal chromosomal diseases.

## Introduction

The development of induced pluripotent stem cells (iPSCs) offers unprecedented opportunities for life sciences, drug development, and cell therapy (Takahashi and Yamanaka, 2006; Takahashi et al., 2007). Human iPSCs have been established from somatic cells in many patients who suffered from various genetic diseases (Park et al., 2008; Hayashi, 2017). These patient-derived iPSCs have been widely utilized for recapitulating pathogenesis *in vitro*, thereby contributing to disease modeling and drug development (Matsumoto et al., 2013; Hayashi et al., 2016).

Utilizing patient-derived iPSCs for abnormal chromosomal diseases has several advantages. First, animal disease modeling is often inappropriate to evaluate abnormal chromosomes due to the structural difference among species. Second, it is hard to apply simple genome editing technology in cells or individuals for abnormal chromosomes. Third, since iPSCs are pluripotent, patient-derived iPSCs can develop disease models with multiple cell and tissue types, which are typically seen in the symptoms of chromosomal diseases.

Thus, patient-derived iPSCs carrying chromosomal abnormality are valuable as cellular bioresources. In this review, we introduce the studies of abnormal chromosomal diseases using patient-derived iPSCs, focusing on pluripotent state and neural lineages. We also discuss the technological advances in chromosomal manipulations toward establishing experimental models and future therapeutic methods.

## Studies of Abnormal Chromosomal Diseases Using Patient-Specific iPSCs

We show the summary list of studies using abnormal chromosomal disease-specific iPSC lines in Table 1.

**Table 1:** Summary list of studies using abnormal chromosomal disease-specific iPSC lines.

Disease name	Genomic features	Major symptoms	Major cellular phenotypes revealed by studies using disease-specific iPSCs	References
Down Syndrome	Trisomy 21	Physical growth delays, intellectual disability, characteristic facial features, poor immune function, congenital heart diseases, epilepsy, leukemia, thyroid diseases, and mental illnesses.	Abnormal chromosome location in a nucleus; abnormal synaptogenesis and neuronal excitability; increased apotosis and downregulation of forehead developmental genes in neural progenitor cells; an abnormal neural differentiation; reduced synaptic activity; decreased levels of synaptogenic molecules and increased levels of reactive oxygen species in astroglia;	Weick et al. (2013), Chen et al. (2014), Hibaoui et al. (2014), Halevy et al. (2016), Dashinimaev et al. (2017), Omori et al. (2017), Araujo et al. (2018), Gonzales et al. (2018), Mizuno et al. (2018), Real et al. (2018) and Ponroy Bally et al. (2020)
Turner Syndrome	Monosomy X	Specific dysmorphic stigmata, short stature, hypogonadism, and renal dysfunctions, cardiac diseases, skeletal defects, endocrine failure, and metabolic deficiency	Lower expression of placental genes in the genomic pseudoautosomal region	Li et al. (2012), Zhang et al. (2013), Dominguez et al. (2014), Parveen et al. (2017); Luo et al. (2018) and Lu et al. (2019)
Klinefelter Syndrome	Sex chromosome trisomy	Tall stature, reduced muscle tone, and hypogonadism	Abnormal gene expression associated with KS symptoms	Ma et al. (2012), Shimizu et al. (2016) and Panula et al. (2019)
Angelman Syndrome	Maternal dysfunction of chromosome 15q11-q13 imprinted region	A small head and a specific facial appearance, severe intellectual disability, developmental disability, speaking problems, balance and movement problems, seizures, and sleep problems	Abnormal DNA methylation status and neuronal maturation defects	Chamberlain et al. (2010), Stanurova et al. (2016), Fink et al. (2017), Takahashi et al. (2017), Neureiter et al. (2018), Pólvora-Brandão et al. (2018) and Niki et al. (2019)
Prader-Willi Syndrome	Paternal dysfunction of chromosome 15q11-q13 imprinted region	Developmental delay, obesity and type 2 diabetes, intellectual impairment and behavioral problems, a narrow forehead, small hands and feet, short height, light skin and hair, infertility	High DNA methylation levels in the imprinting center of the maternal allele; abnormal adherens junctions and apical-basal polarity in neural progenitors	Chamberlain et al. (2010), Yang et al. (2010), Martins-Taylor et al. (2014), Burnett et al. (2016, 2017), Okuno et al. (2017), Langouët et al. (2018) and Soeda et al. (2019)
DiGeorge Syndrome	Heterozygous 22q11.2 deletion syndrome	Congenital heart problems, characteristic facial features, poor immune function, developmental delay, learning problems, cleft palate, kidney dysfunctions, and hearing loss	Reduced size of neurospheres; lower neural differentiation capacity, poor neurite outgrowth, poor neural migration, and an abnormal transition in the neurogenic-to-gliogenic competence	Zhao et al. (2015), Lin et al. (2016) and Toyoshima et al. (2016)
Miller-Dieker Syndrome	Heterozygous deletion of chromosome 17p13.3	Lissencephaly	Neural cell migration defect, reduced size, and mitotic defects associated with a switch from symmetric to asymmetric cell division of ventricular zone radial glia	Bershteyn et al. (2017) and Iefremova et al. (2017)
Phelan-McDermid Syndrome	Heterozygous deletion of chromosome 22q13.3	Developmental delay, intellectual disability, and an increased risk of autism spectrum disorders (ASDs)	Decreased SHANK3 expression and defects in excitatory synaptic transmission	Shcheglovitov et al. (2013)
16p11.2 CNV change	Deletion or duplication of 16p11.2	Neurodevelopmental diseases, including macrocephaly, ASD and schizophrenia	Changes in cell body size and dendrite length, decreased synaptic density	Deshpande et al. (2017)
Williams Syndrome	Deletion of chromosome 7q11.23 region	Intellectual disability, specific personality traits, characteristic facial features, and cardiovascular problems	Abnormal neurogenic commitment from WS-iPSCs	Chailangkarn et al. (2016) and Lalli et al. (2016)

### Down Syndrome (Chromosome 21 Trisomy)

Down syndrome (DS) is a genetic disease caused by a third copy of chromosome 21 and is the most frequent chromosome abnormality, occurring in about 1 in 1,000 (Mai et al., 2019). DS leads to physical growth delays, intellectual disability, characteristic facial features, poor immune function, congenital heart diseases, epilepsy, leukemia, thyroid diseases, and mental illnesses. The elucidation of the pathological mechanisms has been hampered by the lack of experimental models that contain the chromosome 21 trisomy in various cell types. To overcome this limitation, many groups have generated iPSCs and ESCs from DS patients.

These DS-iPSCs were firstly examined in their pluripotent state. In the case of a pair of maternal chromosomes in DS-iPSCs, the homologous copies of chromosomes tended to form an adjacent pair and to locate relatively inside in a nucleus (Omori et al., 2017). Transcriptional profiling of these DS-iPSCs showed the specific effects of the pair of maternal chromosomes in trisomy 21 on gene expression patterns. These results suggested the pathological phenotypes of DS might be contributed by topological interaction between these homologous chromosomes as well as the increased number of human chromosome 21. DS-iPSCs showed the misexpression of neural genes, such as Microtubule-Associated Protein 2 (*MAP2*), Glutamate [NMDA] receptor subunit 3A (*GRIN3A*), Gamma-aminobutyric acid receptor subunit alpha-2 (GABRA2), and Stathmin 2 (*STMN2*) as well as the increased gene expression produced from chromosome 21 trisomy (Gonzales et al., 2018). These results suggested that the chromosome 21 trisomy in iPSCs might disturb the maintenance of pluripotency, but not intrinsically limit neuronal differentiation.

When DS-ESCs were differentiated into neural progenitor cells (NPCs), these NPCs displayed increased apoptosis and downregulation of forehead developmental genes (Halevy et al., 2016). It was also found that *RUNX1* is a key transcription regulator in DS-ESC-NPCs for neural differentiation (Halevy et al., 2016). In these teratomas generated from DS-iPSCs injected intramuscularly into immunodeficient mice, ectodermal tissues were largely absent. When DS-iPSCs were differentiated into neural lineages *in vitro*, the architecture and density of neurons, astroglia, and oligodendrocytes were abnormal together with the misexpression of neuronal genes (Hibaoui et al., 2014). Excitatory and inhibitory synapses in DS-iPSCs-cortical neurons displayed reduced synaptic activity (Weick et al., 2013). Transplantation of DS-iPS-derived cortical neurons into the adult mouse cortex showed increased synaptic stability and reduced oscillation in transplants (Real et al., 2018). DS is associated with an increased risk of Alzheimer’s disease (AD). DS-neurons showed up-regulated expression of the *APP* (Amyloid precursor protein) gene and increased secretion and accumulation of amyloid-β (Aβ) granules made of Abeta42 pathological isoform (Dashinimaev et al., 2017). Additionally, expression levels of AD-associated genes, such as *BACE2*, *RCAN1*, *ETS2*, and *TMED10*, were increased. These findings suggested DS-neurons might recapitulate cellular signs of AD and could be useful models for studying this DS-associated AD subtype. Astroglia from DS-iPSCs produced decreased levels of synaptogenic molecules, thrombospondins 1 and 2 (TSP-1 and TSP-2), and increased levels of reactive oxygen species. Conditioned medium collected from the culture of DS-astroglia invoked neural cell toxicity and failed to promote the maturation of voltage-gated sodium and potassium ion channels and synapse formation in normal and DS neurons. Transplanted DS-astroglia also did not promote neural development in the developing brain of immunodeficient mice (Chen et al., 2014). DS-astroglia also exhibited abnormal synaptogenesis and neuronal excitability with transcriptomic and epigenetic changes in genes associated with neurodevelopmental, cell adhesion and extracellular matrix functions (Araujo et al., 2018; Mizuno et al., 2018; Ponroy Bally et al., 2020). Together, DS-iPSCs and their differentiated neural derivatives recapitulated various cellular defects that were consistent with various symptoms in DS patients and might enable the discovery of the underlying pathology and the development of treatments for DS.

### Aneuploidy of Sex Chromosomes: Turner Syndrome and Klinefelter Syndrome

X chromosome monosomy is the most frequent genetic aberration in about 2% of human conceptions; however, 99% of the conceptions are spontaneously aborted. Those who survive after birth, as called Turner syndrome (TS) patients, are likely to be suffered from specific dysmorphic stigmata, short stature, hypogonadism, and renal dysfunctions, cardiac diseases, skeletal defects, endocrine failure, and metabolic deficiency. Several studies have successfully generated TS-iPSCs (Li et al., 2012; Parveen et al., 2017; Luo et al., 2018; Lu et al., 2019). TS-iPSCs were examined their global gene expression patterns (Zhang et al., 2013). They differentiated into various somatic cells in embryoid bodies (EBs) but displayed lower expression of placental genes, *ASMTL*, *PPP2R3B*, and *CSF2RA* in the genomic pseudoautosomal region. These findings suggested that abnormal organogenesis and embryonic lethality in TS might not be caused by an abnormal tissue-specific differentiation capacity, but might be caused by other abnormalities including impaired placental development (Li et al., 2012). TS-iPSCs were also able to form germ-cell-like cells *in vivo* through xenotransplantation into mice (Dominguez et al., 2014). These results suggested that two intact X chromosomes might not be essential for human germ cell differentiation at its initial phase.

Klinefelter syndrome (KS), or two or more X chromosomes in males (XXY), occurs in about 1 in 1,000 (Bojesen et al., 2003). Because X chromosome inactivation (XCI) occurs in KS patients heterogeneously, they develop a variety of clinical symptoms, including tall stature, reduced muscle tone, and hypogonadism. The KS pathophysiology remains elusive due to the lack of experimental models. Some studies reported the successful generation of KS-iPSCs with a karyotype of 47, XXY (Ma et al., 2012; Shimizu et al., 2016; Panula et al., 2019). Although XCI occurs in KS-iPSCs, their transcriptome profile identified abnormally expressed genes associated with KS symptoms. These KS-iPSCs should be useful in revealing the mechanisms of human XCI and fertility.

### Angelman Syndrome (AS) and Prader-Willi Syndrome (PWS)

Angelman Syndrome (AS) and Prader-Willi Syndrome (PWS) are two distinct neurological diseases caused by the dysfunction of the genes located on chromosome 15q11-q13 imprinted region. AS arises from dysfunction of the ubiquitin-protein ligase E3A (UBE3A), while the responsible genetic defects in PWS remain elusive. Several research groups have successfully generated AS-iPSCs and demonstrated abnormal DNA methylation status and neuronal maturation defects (Chamberlain et al., 2010; Sakurai et al., 2016; Fink et al., 2017; Takahashi et al., 2017; Neureiter et al., 2018; Pólvora-Brandão et al., 2018; Niki et al., 2019). These phenotypes could be reversed by recovering paternal *UBE3A* expression by the treatment with a topoisomerase inhibitor, topotecan (Fink et al., 2017). Some research groups have also generated iPSCs from PWS patients (Chamberlain et al., 2010; Yang et al., 2010; Martins-Taylor et al., 2014; Burnett et al., 2016, 2017; Okuno et al., 2017; Langouët et al., 2018; Soeda et al., 2019). These PWS-iPSCs showed high DNA methylation levels in the imprinting center of the maternal allele. Also, maternally expressed genes in the *DLK1-DIO3* imprinting loci on chromosome 14, which was regulated by IPW, a long non-coding RNA located in chromosome 15q11-q13 imprinted region, was up-regulated (Stelzer et al., 2014). These results suggested that a part of PWS phenotypes might be caused by the dysregulation of imprinted loci distinct from the 15q11-q13 imprinted region. Another study showed that PWS-iPSC-derived neural progenitors exhibited abnormal adherens junctions and apical-basal polarity (Yoon et al., 2014). These findings suggested that AS-and PWS-iPSCs were useful tools to study genetic imprinting diseases.

### DiGeorge Syndrome (DGS)

DiGeorge syndrome (DGS), also known as 22q11.2 deletion syndrome or CATCH 22, causes congenital heart problems, characteristic facial features, poor immune function, developmental delay, learning problems, cleft palate, kidney dysfunctions, and hearing loss. Several groups have generated DGS patient-derived iPSCs carrying 22q11.2 deletion. Neurospheres, a culture system composed of floating clusters of neural stem cells, derived from the DGS-iPSCs showed reduced size, lower neural differentiation capacity, poor neurite outgrowth, poor neural migration, and an abnormal transition in the neurogenic-to-gliogenic competence. Transcriptomic profile in DGS-neurons also revealed significant differences in many genes outside of the deleted region as well as gene expression reduction in the 22q11.2 region. Key pathways and gene ontology, such as apoptosis, cell cycle and survival, and MAPK signaling, were uncovered by functional enrichment and network analysis on the differentially expressed genes (Lin et al., 2016). The global miRNA profiling in these neurospheres showed decreased expression of the miR-17/92 cluster and miR-106a/b, which were known to control cell proliferation (Toyoshima et al., 2016). Many differentially expressed miRNAs were also detected, including miRNAs located in the 22q11.2 region. Genes involved in neurological diseases were predicted to be the targets for the differentially expressed miRNAs by the pathway and gene ontology enrichment analysis (Zhao et al., 2015).

### Neurodevelopmental Diseases Associated With Chromosomal Abnormalities

Miller-Dieker syndrome (MDS) is caused by a heterozygous deletion of chromosome 17p13.3 and leads to lissencephaly (meaning “smooth brain”), a lack of development of brain folds and grooves because of defective neuronal migration. Patient-specific forebrain-type organoids were generated from MDS-iPSCs to investigate pathological changes associated with MDS (Bershteyn et al., 2017; Iefremova et al., 2017). MDS-organoids showed cell migration defect, reduced size, and mitotic defects associated with a switch from symmetric to asymmetric cell division of ventricular zone radial glia. The cell migration defect was rescued when the chromosomal deletion in MDS-iPSCs was corrected as uniparental disomy (UPD; the details of the methods will be explained later; Bershteyn et al., 2014). Mitotic defects in outer radial glia, which could not be seen in the experimental rodent model of lissencephaly, was observed (Bershteyn et al., 2017). In another study, the treatment of a WNT beta-catenin signaling activator, CHIR99021, rescued cell division modes, and organoid growth defects (Iefremova et al., 2017).

Phelan-McDermid syndrome (PMDS) is caused by a heterozygous deletion of chromosome 22q13.3 and leads to developmental delay, intellectual disability, and an increased risk of autism spectrum disorders (ASDs). Among the genes in the deleted region, *SHANK3*, which encodes a multidomain scaffold protein in the postsynaptic density, is the candidate responsible gene for the neurological symptoms. One study reported the generation of PMDS-iPSCs to recapitulate neuronal symptoms. PMDS-neurons exhibited decreased SHANK3 expression and defects in excitatory α-amino-3-hydroxy-5-methyl-4-isoxazolepropionic acid (AMPA)-and N-methyl-D-aspartic acid (NMDA)-mediated synaptic transmission. These defects were rescued by reactivating SHANK3 expression or by treating with insulin-like growth factor 1 (IGF1), which had been previously reported to increase synaptic transmission (Shcheglovitov et al., 2013).

A change of copy number variation (CNV) in the 16p11.2 region, which is caused by both deletion (16pdel) and duplication (16pdup), is associated with neurodevelopmental diseases, including macrocephaly, ASD and schizophrenia. One research group generated iPSCs from 16pdel and 16pdup patients and differentiated them into neurons to identify causal cellular phenotypes underlying neurological symptoms. 16pdel neurons showed increased cell body size and dendrite length, while 16pdup neurons showed decreased cell body size and dendrite length. Notably, both 16pdel and 16pdup neurons showed decreased synaptic density (Deshpande et al., 2017). These results suggested the 16p11.2 region might regulate brain size and neuronal connectivity distinctively.

Williams syndrome (WS) is caused by a deletion of chromosome 7q11.23 region, which contains approximately 28 genes. WS patients are suffered from intellectual disability, specific personality traits, characteristic facial features, and cardiovascular problems. Two research groups generated patient-derived WS-iPSCs and differentiation them into neurons (Chailangkarn et al., 2016; Lalli et al., 2016). Both studies showed that the global transcriptional profile of WS-iPSC-derived neurons confirmed the expression changes of the deleted genes and that NPCs from WS-iPSCs exhibited abnormal neurogenic commitment; however, the rate of cell cycle and apoptosis in these cells was somewhat inconsistent between these studies. The first study focused on Bromodomain Adjacent To Zinc Finger Domain 1B (*BAZ1B*) gene, which is deleted in WS and encodes an ATP-dependent chromatin remodeling protein. Knocking down BAZ1B mimicked transcriptional and neural differentiation defects as seen in WS-derived cells. These defects were rescued by antagonizing Wnt signaling because this signaling pathway was identified in target genes of BAZ1B revealed by ChIP-seq analysis (Lalli et al., 2016). The second study focused on Frizzled-9 (*FZD9*), which was also deleted in WS and encodes transmembrane receptors for Wnt proteins. Reduced cell viability was rescued by a GSK3 inhibitor, which acts as a Wnt signaling activator. Although these studies seemingly demonstrated contradictory findings, both emphasized the role of Wnt signaling in WS pathology.

## Discussion: Chromosomal Manipulation Technologies

**Figure 1:**
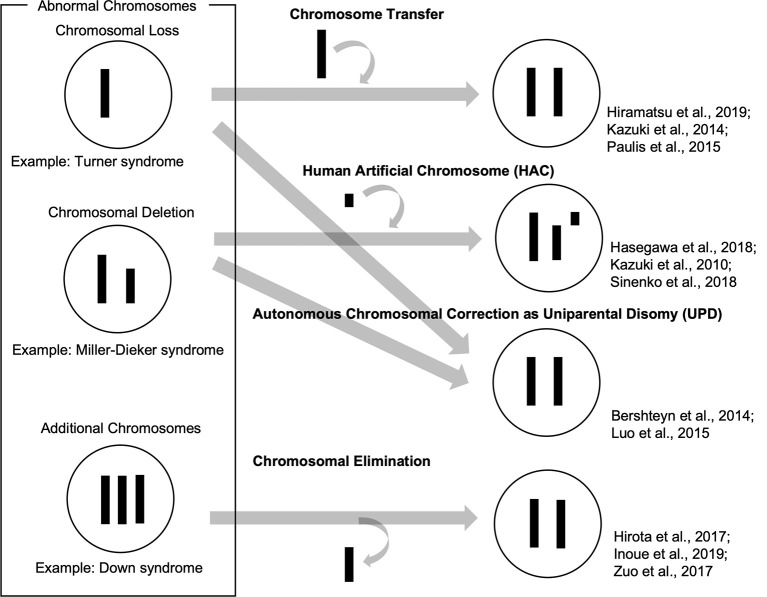
Schemes of current technologies for chromosomal manipulation.

So far, we have reviewed how patient-derived iPSCs have been used as valuable tools to recapitulate abnormal chromosomal diseases to elucidate disease mechanisms and develop potential therapies. To enhance, the technological advances of chromosomal manipulation are keys to improved usability of patient-derived iPSCs carrying abnormal chromosomes and the development of disease models and future therapeutic methods. In this chapter, we discuss current technologies for chromosomal manipulation (illustrated in a scheme in Figure 1).

### Chromosome Transfer

Chromosome transfer can be achieved by extracting a chromosome from a cell and inserting it into another cell. Human pluripotent stem cells carrying trisomy 8, 13, 18, and 21 were generated by a single chromosome insertion *via* microcell-mediated chromosome transfer. Global trisomic expression levels were confirmed by microarray analysis in each cell line (Kazuki et al., 2014; Hiramatsu et al., 2019). Another study reported successful chromosome transfers by replacing an X chromosome carrying a mutation in the *Hprt* gene with a normal one without the mutation in mouse embryonic stem cells (mESCs), which enables HAT (hypoxanthine-aminopterin-thymidine) selection. They also transferred a Y chromosome to rescue the defective Hprt gene, which is common in sex chromosomes. These mESC clones, which were transferred with a sex chromosome, maintained their pluripotency and genomic integrity and contributed to chimera formation (Paulis et al., 2015). Although it has not been directly applied, these studies suggested that intact chromosomes could be transferred into patient-derived iPSCs carrying abnormal chromosomes. This approach might be used to cure various disorders with chromosomal loss or deletions.

### Human Artificial Chromosome (HAC)

Human artificial chromosome (HAC) vectors hold the potential to rescue diseases associated with chromosomal deletion and loss since HAC could hold DNA inserts of any size in principle. Several studies showed the successful integration of HAC vectors into iPSCs (Kazuki et al., 2010; Hasegawa et al., 2018; Sinenko et al., 2018). One study showed that a HAC vector carrying whole dystrophin (*DMD*) genomic sequence rescued dystrophin expression of Duchenne muscular dystrophy (DMD) in patient-specific iPSCs (Kazuki et al., 2010). These results suggested that the HAC vectors containing defective genes could be valuable tools to rescue the phenotypes in patient-specific iPSCs carrying specific gene deletions. Although there has been no direct evidence that HAC vectors can be used for abnormal chromosomal diseases to date, this strategy could be applied to achieve gene and cell therapies for abnormal chromosomal diseases. However, it is challenging that cloning desired long regions of the human genome into HAC and transferring them into iPSCs and other human somatic cells.

### Autonomous Chromosomal Correction as Uniparental Disomy (UPD)

Several studies demonstrated that abnormal chromosomes were autonomously corrected in iPSCs. A Ring chromosome is an aberrant chromosome whose ends have fused to form a ring. We previously generated iPSCs from patients’ somatic cells carrying ring chromosomes with terminal deletions and identified that these reprogrammed iPSCs lost ring chromosomes and duplicated the intact homologous chromosome. The resulting iPSCs carried a pair of identical chromosomes, UPD (Bershteyn et al., 2014). These karyotypically normal UPD iPSCs proliferated at a better/improved rate over co-existing subpopulations carrying ring chromosomes or monosomy. These patient-derived iPSCs free from the original chromosomal abnormality were efficiently isolated. Another study reported that some iPSCs generated from somatic cells in TS patients, carrying X monosomy acquired XX UPD karyotype. The XX-UPD iPSCs showed XCI, better pluripotent stem cell morphology, and higher mitotic rate than uncorrected ones (Luo et al., 2015). These studies demonstrated human iPSCs were a useful model for the investigation of mechanisms that control the number and behaviors of chromosomes during development/differentiation. Although the UPD phenomenon occurs spontaneously, it could serve as a model strategy for the development of innovative methods aimed at targeting large-scale chromosomal deletions or entire chromosome loss.

### Chromosome Elimination

Some studies demonstrated spontaneous or targeted chromosomal elimination in iPSCs that carry extra chromosomes. A study showed that iPSCs from XXY and XYY trisomy mice lost an extra sex chromosome during the reprogramming process (Hirota et al., 2017). Resulting disomic XY iPSCs successfully differentiated into male germ cell lineages and functional sperms which produced fertile offspring with normal chromosomes. Other research groups demonstrated to eliminate Y chromosomes in male mESCs using genome editing technology by removing the centromere or shredding the chromosome arm (Adikusuma et al., 2017; Zuo et al., 2017). Also, several studies demonstrated to produce a targeted autosome loss in human TS-iPSCs with trisomy 21 and KS-iPSCs with sex chromosome trisomy (Hirota et al., 2017; Zuo et al., 2017; Inoue et al., 2019). Another method was reported to use X-Inactive Specific Transcript (XIST) RNA, which was normally located in the X chromosome and acts as a major effector of the X-inactivation process. Knocking-in XIST locus in the extra chromosome 21 of DS patient-derived cells could induce chromosome-wide silencing of the targeted chromosome (Jiang et al., 2013; Chiang et al., 2018; Czermiński and Lawrence, 2020). Thus, spontaneous and targeted chromosome elimination or silencing can offer new approaches to enhance the usability of disease models with abnormal chromosomes and to provide potential therapeutic methods for diseases involving additional chromosomes.

## Conclusions

We have discussed the usability of patient-derived iPSCs carrying chromosomal abnormality. To establish platforms to develop therapeutics for these diseases related to chromosomal abnormality, patient-derived iPSCs are highly beneficial. Disease-specific iPSCs in major cell banks worldwide can be searched for using ICSCB (Integrated Collection of Stem Cell Bank) data^1^ by MIACARM (Minimum Information About a Cellular Assay for Regenerative Medicine; Sakurai et al., 2016). We might apologize for not referring to some studies using patient-derived iPSCs on rare chromosomal abnormalities due to the word limit. Although many of these studies are still immature, they have generated patient-derived iPSCs that will serve as valuable bioresources/models for the investigation into the pathogenesis of abnormal chromosome disease with the potential of developing treatment strategies. We have also introduced current technologies in chromosomal manipulation. For the future clinical application of these technologies, we need to discuss adequately to reach social consensus on ethical conflict/controversy with such genetic manipulations.

## Author Contributions

YH contributed to the conception and design of the study. YH, MT, and MM-T wrote sections of the manuscript. All authors contributed to the article and approved the submitted version.

## Conflict of Interest

The authors declare that the research was conducted in the absence of any commercial or financial relationships that could be construed as a potential conflict of interest.

## Footnotes

^1^https://icscb.stemcellinformatics.org/
